# Insulin Deficiency Exacerbates Muscle Atrophy and Osteopenia in *Chrebp* Knockout Mice

**DOI:** 10.3390/ijms262311672

**Published:** 2025-12-02

**Authors:** Chihiro Ushiroda, Mioko Ito, Risako Yamamoto-Wada, Kanako Deguchi, Shihomi Hidaka, Toshinori Imaizumi, Yusuke Seino, Atsushi Suzuki, Daisuke Yabe, Katsumi Iizuka

**Affiliations:** 1 Department of Clinical Nutrition, Fujita Health University, Toyoake 470-1192, Japan; chihiro.ushiroda@fujita-hu.ac.jp (C.U.); ito@nagoya-aoi.ac.jp (M.I.); risako.wada@fujita-hu.ac.jp (R.Y.-W.); kanasakuran@gmail.com (K.D.); 2 Department of Endocrinology and Metabolism, Fujita Health University, Toyoake 470-1192, Japan; sakai220@fujita-hu.ac.jp (S.H.); seinoy@fujita-hu.ac.jp (Y.S.); aslapin@fujita-hu.ac.jp (A.S.); 3 Department of Diabetes, Endocrinology and Nutrition, Kyoto University Graduate School of Medicine, Kyoto 606-8507, Japan; imaizumits@kuhp.kyoto-u.ac.jp (T.I.); ydaisuke@kuhp.kyoto-u.ac.jp (D.Y.); 4 Food and Nutrition Service Department, Fujita Health University Hospital, Toyoake 470-1192, Japan

**Keywords:** ChREBP, bone mineral density, grip strength, streptozocin, muscle atrophy

## Abstract

Type 1 diabetes mellitus is a major risk factor for both sarcopenia and osteoporosis, primarily due to the body’s inability to utilize glucose as a result of insulin deficiency. Impairments in insulin and glucose signaling can accelerate the decline in muscle and bone health. To investigate this interaction, we examined whether insulin deficiency exacerbates muscle and bone deterioration in *Chrebp* knockout (KO) mice. Male wild-type (WT) and KO mice, aged 18 weeks, were intraperitoneally treated with 200 mg/kg BW streptozotocin (STZ), which selectively destroys pancreatic beta cells, thereby inducing insulin deficiency. Two weeks after STZ administration, compared with STZ-treated WT mice, STZ-treated KO mice presented significantly greater reductions in body weight and gastrocnemius muscle weight (BW: WT-vehicle vs. WT-STZ; 2.58 [−1.23, 6.39] (*p* = 0.21); KO-vehicle vs. KO-STZ: 8.03 [5.23, 10.82]; GA muscle: WT vehicle vs. WT STZ: 0.084 [0.047, 0.12], *p* < 0.0001; KO vehicle vs. KO STZ: 0.084, [0.047, 0.12], *p* < 0.0001). The decrease in grip strength caused by STZ administration was greater in the KO mice than in the WT mice (mean differences [95% CIs]: WT vehicle—WT STZ, 49.6. [0.9, 98.4], *p* = 0.046; WT STZ—KO STZ: 71.40 [29.1, 113.7], *p* = 0.0059; KO vehicle—KO STZ: 84.3 [51.9, 116.8], *p* = 0.0003). Consistent with these findings, STZ administration reduced IGF-1 expression and increased atrogin mRNA levels, with the highest levels in STZ-treated KO mice. In skeletal muscle, the changes in IGF-1 and Atrogen induced by STZ administration were significantly greater in the KO group than in the WT group (IGF-1: WT vehicle—WT STZ: 0.19 [−0.072, 0.46], *p* = 0.17; KO vehicle—KO STZ: 0.79 [0.53, 1.06], *p* < 0.0001; Atrogen: WT vehicle—WT STZ: −2.7 [−3.01, −2.29], *p* < 0.0001; KO vehicle—KO STZ: −3.35 [−3.71, −2.99], *p* < 0.0001). The BMD in the *Chrebp*-deficient group was greater than that in the wild-type group (WT vehicle—KO vehicle: −5.2 [−8.4, −1.9], *p* = 0.0014); however, the administration of STZ significantly decreased the BMD only in the KO group (WT vehicle—WT STZ: *p* = 0.45, KO vehicle—KO STZ: 7.2 [3.9, 10.4], *p* < 0.0001). These results suggest that *Chrebp* deficiency combined with insulin deficiency aggravates sarcopenia and osteoporosis risk. Therefore, insulin and glucose signals are important for maintaining muscle and bone mass and function. However, further studies are needed to elucidate the mechanisms by which ChREBP deletion and insulin deficiency cause osteosarcopenia.

## 1. Introduction

Type 1 diabetes mellitus (T1DM) is predominantly caused by autoimmune processes and requires lifelong insulin therapy [1]. Pancreatic islet transplantation and hepatocyte therapy have been attempted in recent years as treatments for T1DM, but these treatments are still under development [2,3]. Recent advances in insulin therapies, such as continuous subcutaneous insulin infusion (CSII), have markedly improved blood glucose management compared with previous methods [4,5,6]. A current primary concern for older adults, particularly those with chronic conditions, is the prevention of diseases such as frailty, sarcopenia, and osteoporosis that adversely affect healthy life expectancy [7,8,9]; individuals with T1DM also face this concern.

Streptozotocin (STZ)-induced diabetes is the most widely used chemical model of type 1 diabetes mellitus (T1DM) in experimental animals [10]. Because STZ is selectively taken up by pancreatic β-cells via GLUT2 and induces DNA alkylation, NAD^+^ depletion, and mitochondrial dysfunction, it results in rapid and profound insulin deficiency, mimicking the cardinal metabolic features of T1DM [11]. STZ-induced diabetes represents a nonautoimmune, direct β-cell destruction model, allowing researchers to study the consequences of insulin deficiency independent of immune mechanisms [12]. Thus, the STZ model is best positioned as a robust, accessible, and reproducible tool for investigating the metabolic and organ-level consequences of severe insulin deficiency rather than as a model of autoimmune pathogenesis.

In the context of osteoporosis, the pathophysiological differences between type 1 and type 2 diabetes are notable. T1DM is characterized by a substantial reduction in bone mineral density, whereas alterations in bone quality are observed in T2DM [13,14,15]. Furthermore, individuals with lower body weights are at greater risk of osteoporosis than those who are overweight. In addition, current evidence indicates that inadequate blood glucose regulation elevates the risk of sarcopenia, regardless of whether an individual has T1DM or T2DM [16].

Osteosarcopenia is a muscle–bone unit dysfunction in which osteoporosis and sarcopenia occur simultaneously, and it is characterized by a synergistic increase in the risk of falls, fractures, and frailty when both conditions coexist [1]. In addition to elderly individuals and patients in hospitals or nursing facilities, the prevalence of osteosarcopenia increases to 20–30% in people with type 2 diabetes [17,18]. Moreover, osteosarcopenia occurs when insulin levels are low in patients with type I diabetes mellitus [19,20]. Insulin deficiency leads to decreased amino acid uptake, reduced protein synthesis due to decreased mTOR activity, and increased protein breakdown due to FOXO activation, resulting in reduced muscle mass and muscle strength. Insulin deficiency also causes decreased bone formation and increased bone resorption due to the activation of osteoclasts [21,22]. In addition, abnormalities in the muscle–bone–adipose axis are known as a pathology of osteosarcopenia caused by insulin deficiency [21,22]. The amount of bone marrow fat also increases, which is believed to lead to decreased muscle strength and bone mass. These changes create a vicious cycle in the interorgan functional network (muscle–bone–fat axis). In bone, muscle-derived IL-6 promotes bone formation, whereas myostatin promotes bone resorption. It is also known that a decrease in muscle strength itself reduces mechanical loading on bone and decreases bone formation. TNF1α/IL-6 secreted from adipocytes activates osteoclasts and promotes muscle protein breakdown [21,22]. Therefore, although its expression in bone remains unclear, systemic deletion of Chrebp may affect the muscle–bone–adipose axis and potentially impact the functions of both bone and muscle. In a previous study, we reported that deletion of Chrebp alone results in a reduction in grip strength but an increase in bone density [23]. Moreover, the introduction of a low-protein diet led to decreased grip strength, reduced muscle mass, and decreased bone density in *Chrebp* knockout mice. Therefore, it is possible that not only insufficient insulin action but also abnormalities in glucose signaling may cause dysfunction of bones and muscles.

Glucose and insulin coordinately regulate various physiological functions, including fatty acid synthesis by the liver [24,25,26,27,28,29,30,31,32,33,34,35,36]. Carbohydrate response element binding protein (*Chrebp*) is a transcription factor that is responsible for regulating de novo lipogenesis and converts sugars into fatty acids; it is expressed in the liver, adipocytes, skeletal muscle, small intestine, adrenal glands, and pancreatic β-cells [24,25,26,27,28,29,30,31,32,33,34,35,36]. The main effects of Chrebp include converting excess carbohydrates into triglycerides and storing them in lipid-synthesizing organs such as the liver and adipose tissue, as well as being involved in fructose metabolism in the small intestine and liver [24,25,26,27,28,29,30,31,32,33,34,35,36]. Glucose is metabolized under the action of insulin, and its metabolic products (G6P, X5P) activate Chrebp [28]. Therefore, because insulin is an essential signal required for the initial uptake and metabolism of glucose into cells, the effects of blocking insulin signaling are expected to surpass those of blocking glucose signaling. Since glucose and insulin both regulate certain metabolic pathways in common, blocking both insulin signaling and glucose signaling in muscle tissue is anticipated to lead to decreased muscle strength. On the other hand, as regulatory genes for glucose and insulin can act either positively or negatively, we considered that either glucose blockade or insulin blockade—whichever effect is stronger—would manifest in bone tissue. On the basis of the above findings, we hypothesized that blocking both insulin and glucose signals, e.g., STZ treatment and *Chrebp* gene deletion, would result not only in weight loss but also in reductions in adipose tissue, muscle mass, and bone mass.

In this study, we sought to clarify the roles of insulin and glucose signaling in maintaining bone and skeletal muscle function by using *Chrebp* knockout (KO) mice and a streptozotocin (STZ)-induced insulin deficiency model. By comparing normal wild-type (WT) models with STZ-treated WT mice, we can assess the effects of insulin deficiency on bones and muscles. Moreover, comparing WT mice with KO mice allows us to evaluate the impact of *Chrebp* deficiency, which is downstream of the glucose signal, on bones and muscles. By comparing vehicle-treated WT mice with vehicle-treated KO mice, we elucidated the effects of Chrebp on bones and muscles under various conditions, especially insulin. Additionally, by comparing STZ-treated WT mice with STZ-treated KO mice, we elucidated the effects of Chrebp on bones and muscles under insulin-deficient conditions, highlighting the insulin-independent role of Chrebp. This study provides important insights into the roles of insulin and glucose in maintaining bones and muscles.

## 2. Results

### 2.1. STZ Administration Induces Diabetes Mellitus in WT and KO Mice

In previous studies, we showed that feeding a low-protein diet to *Chrebp* knockout mice led to osteoporosis and muscle weakness. Insulin deficiency also decreases bone density and causes muscle weakness; this study aimed to determine whether reduced glucose signaling due to *Chrebp* deficiency further exacerbates the decline in bone density and muscle strength. We confirmed that both WT and KO mice develop hyperglycemia following STZ administration.

**Figure 1:**
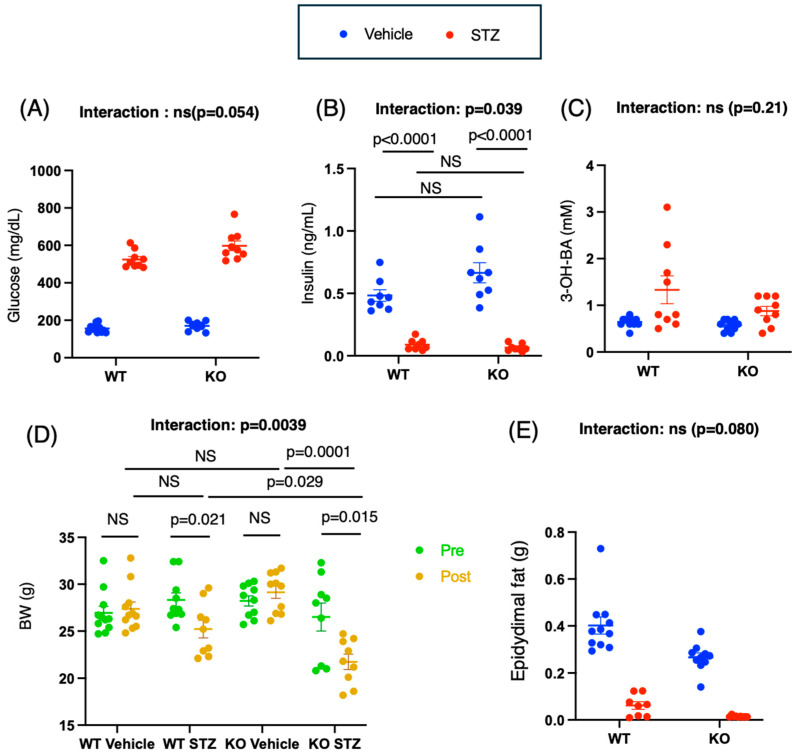
Effects of *Chrebp* deletion and insulin deficiency on blood glucose levels, insulin levels, 3-OHBA levels, and epidydimal fat weight. (**A**) Plasma glucose levels. Two-way ANOVA. Genotype (WT vs. KO): *p* < 0.0001; drug (STZ): *p* = 0.0008; genotype × drug interaction: *p* = 0.054. (**B**) Plasma insulin levels (ng/mL). Two-way ANOVA. Genotype (WT vs. KO): *p* = 0.11; drug (STZ): *p* < 0.0001; genotype × drug interaction: *p* = 0.039. (**C**) 3-Hydroxybutyrate levels. Two-way ANOVA. Genotype (WT vs. KO): *p* = 0.081; drug (STZ): *p* = 0.0018; genotype × drug interaction: *p* = 0.221. Blood parameters were measured in 20-week-old male mice (*n* = 9–11) (vehicle: blue; streptozocin (STZ): red). (**D**) Total body weight (grams). Two-way ANOVA. group: *p* = 0.055; time: *p* = 0.0002; genotype × time interaction: *p* = 0.0039. (**E**) Epididymal fat weight (g). Two-way ANOVA. Genotype (WT vs. KO): *p* = 0.008; drug (STZ): *p* = 0.079; genotype × drug interaction: *p* < 0.0001. *n* = 9–11 for each group (control diet: blue; STZ: red). STZ was administered intraperitoneally to 18-week-old WT and KO mice, and measurements were made two weeks later. The plasma levels were measured via the methods described in the methods section. The data are presented as the means ± standard errors of the means (SEMs) and were analyzed via one-way analysis of variance (ANOVA) with post hoc Tukey’s test for each group (vehicle: blue; STZ: red). Post hoc analyses were performed following two-way ANOVA exclusively for interactions that demonstrated statistical significance. *n* = 9–11, *p* < 0.05. NS, not significant.

As a result of performing a two-way ANOVA on glucose, the main effect of genotype (WT vs. KO) was significant (*p* = 0.0059). The main effect of drug administration was also significant (*p* < 0.0001). On the other hand, the interaction between genotype × drug tended to be significant (*p* = 0.054). Blood glucose levels increased to approximately 600 mg/dL in both the WT and KO groups following STZ administration (mean difference [95% CI]: vehicle-treated WT (WT-vehicle) vs. STZ-treated WT (WT-STZ), −368.6 [−424.6, −312.7] (*p* < 0.0001); vehicle-treated KO (KO-vehicle) vs. STZ-treated KO, −427.8 [−485.0, −370.6] (*p* < 0.0001)) (Figure 1A). The blood glucose levels of the animals in the WT-STZ group were slightly greater than those of the animals in the KO-STZ group (WT-STZ group vs. KO-STZ group: −73.11 [−131.8, −14.4] (*p* = 0.0098)) (Figure 1A).

With respect to insulin, the main effects of the drug (STZ) (*p* < 0.0001) and the genotype × drug interaction (*p* = 0.039) were significant. Consistent with the blood glucose data, insulin levels (ng/dL) decreased following STZ administration (WT-vehicle vs. WT-STZ: 0.39 [0.21, 0.58], *p* < 0.0001; KO-vehicle vs. KO-STZ: 0.60 [0.42, 0.78], *p* < 0.0001) (Figure 1B). The KO group presented a greater decrease in insulin levels following STZ administration.

For 3-OHBA, only the main effect of the drug (STZ) was significant (*p* = 0.0018), whereas the main effects of genotype (*p* = 0.081) and the interaction effect between genotype and drug (*p* = 0.21) were not significant (Figure 1C).

Thus, STZ administration increased blood glucose levels and decreased insulin levels in both WT and KO mice, but ketone (3-OHBA) levels increased only in wild-type mice. The effects of increased blood glucose and decreased insulin secretion caused by STZ were more pronounced in the KO group.

Two-way ANOVA revealed a significant main effect of time (*p* = 0.0002) and a significant interaction effect between time and group (*p* = 0.0039). Two weeks post-STZ treatment, both WT and KO mice presented reduced body weights (mean difference pre-post [95% CI]: WT vehicle: −0.44 [−2.55, 1.67], WT-STZ: 3.10 [0.53, 5.66], KO vehicle: −0.93 [−2.74, 0.88], KO-STZ: 4.77 [1.10, 8.43]) (Figure 1D). At 18 weeks of age, no intergroup differences were observed; however, following STZ administration, the mean differences between the WT-vehicle and WT-STZ, WT-STZ and KO-STZ, and KO-vehicle and KO-STZ groups were 2.58 [−1.23, 6.39] (*p* = 0.21), 4.35 [0.57, 8.13] (*p* = 0.029), and 8.03 [5.23, 10.82] (*p* = 0.0001), respectively (Figure 1D). Thus, the body weight loss effect induced by STZ was significantly pronounced in the KO group. With respect to epididymal fat, two-way ANOVA revealed a significant main effect of genotype (WT vs. KO) (*p* = 0.008) and a significant main effect of drug (STZ) (*p* < 0.0001). The genotype × drug interaction tended to be significant (*p* = 0.080) (Figure 1E). The differences in epididymal fat weight between the STZ-treated WT mice and the STZ-treated KO mice were not significant (*p* < 0.0001) (Figure 1E). These findings suggest that STZ administration led to a reduction in epididymal fat weight in both WT and KO mice, which was attributable to the inherently lower fat content in KO mice. In both STZ-treated WT and STZ-treated KO mice, weight loss and a reduction in epididymal fat were observed alongside hyperglycemia and hypoinsulinemia.

### 2.2. Chrebp Deletion Promotes Muscle Atrophy and Decreases Grip Strength in STZ-Treated Mice

Next, we examined the effects of insulin deficiency and *Chrebp* deletion on the mass and strength of mouse skeletal muscles (GA, tibialis anterior (TA), extensor digitorum longus (EDL), and soles (SOL)).

**Figure 2:**
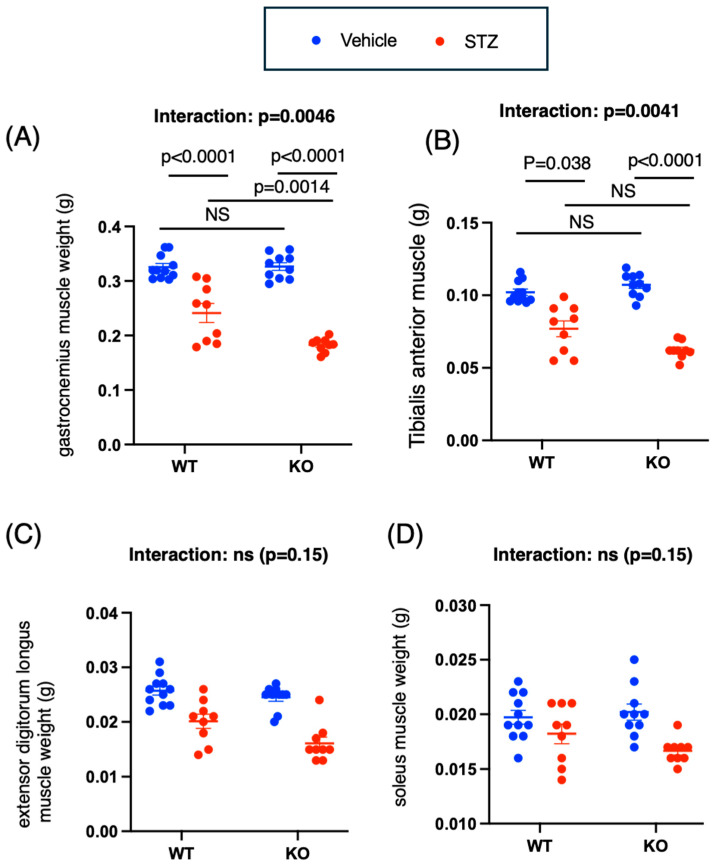

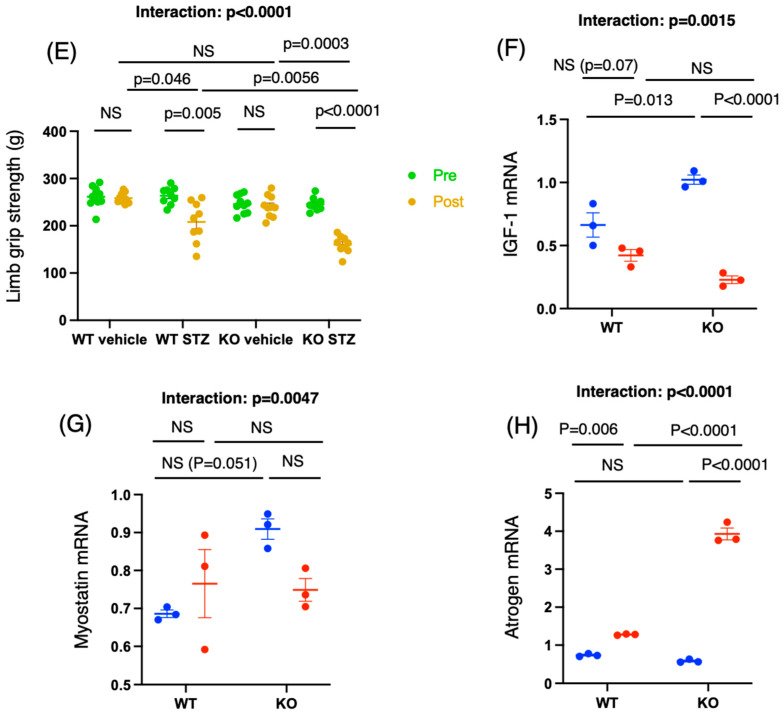
Effects of *Chrebp* deletion and insulin deficiency on muscle weight, grip strength, and muscle mRNA expression in mice. (**A**) Gastrocnemius muscle weight (g). Two-way ANOVA. Genotype (WT vs. KO): *p* = 0.0057; drug (STZ): *p* < 0.001; genotype × drug interaction: *p* = 0.0046. (**B**) Tibialis anterior (TA) muscle weight (g). Two-way ANOVA. Genotype: *p* = 0.15; Drug: *p* < 0.001; genotype × drug interaction: *p* = 0.0041. (**C**) Extensor digitorum longus muscle weight (g). Two-way ANOVA. Genotype (WT vs. KO): *p* = 0.012 drug (STZ): *p* < 0.001; genotype × drug interaction: *p* = 0.17. (**D**) Soleus muscle weight (g). Two-way ANOVA. Genotype (WT vs. KO): *p* = 0.44; drug (STZ): *p* = 0.0008; genotype × drug interaction: *p* = 0.15. (**E**) Limb grip strength (g). *n* = 9–11 for each group (vehicle: blue; STZ: red). Two-way ANOVA. Group: *p* = 0.44; Time: *p* = 0.0008; Group × time interaction: *p* = 0.15. STZ was administered intraperitoneally to 18-week-old WT and KO mice, and experiments were conducted two weeks later. The tissues were removed and weighed. Muscle isolation was performed according to previous reports [27]. The data are presented as the means ± standard errors of the means (SEMs) and were analyzed via two-way analysis of variance (ANOVA) with post hoc Tukey’s test for each group (vehicle: blue; STZ: red). *n* = 9–11, *p* < 0.05. Pre: 18 weeks of age, Post: 20 weeks of age. (**F**) Muscle insulin-like growth factor (Igf-1) mRNA levels. Two-way ANOVA. Genotype (WT vs. KO): *p* = 0.19; drug (STZ): *p* < 0.001; genotype × drug interaction: *p* = 0.0015. (**G**) Muscle myostatin mRNA levels. Two-way ANOVA. Genotype (WT vs. KO): *p* = 0.070; drug (STZ): *p* = 0.44; genotype × drug interaction: *p* = 0.042. (**H**) Muscle androgen mRNA levels. Two-way ANOVA. Genotype (WT vs. KO): *p* < 0.001; drug (STZ): *p* < 0.001; genotype × drug interaction: *p* < 0.001. The anterior tibialis muscles were crushed under liquid nitrogen; mRNA was extracted from the muscle, and cDNA was synthesized as described in the Materials and Methods. Igf-1, myostatin, and atrogin mRNA levels were measured via real-time PCR. The data are presented as the means ± standard errors of the means (SEMs) and were analyzed via two-way ANOVA with post hoc Tukey’s test for each group (vehicle: blue; STZ: red). Post hoc analyses were performed following two-way ANOVA exclusively for interactions that demonstrated statistical significance. *n* = 3, *p* < 0.05. NS, not significant.

According to the results of the two-way ANOVA for GA and TA, there was a significant interaction effect between genotype and drug (*p* = 0.0046) on GA and an interaction effect between genotype and drug (*p* = 0.0041) on TA. The weights of the GA and TA were significantly greater in the STZ-treated WT mice than in the STZ-treated KO mice (mean difference [95% CI]: (WT STZ vs. KO STZ): GA, 0.059 [0.020, 0.098] (*p* = 0.0014); TA, 0.015 [0.0019, 0.027] (*p* = 0.019)) (Figure 2A,B). The GA and TA weights of the STZ-treated KO mice were also lower than those of the vehicle-treated KO mice (mean difference [95% CI]: GA: WT vehicle vs. WT STZ; 0.084 [0.047, 0.12], *p* < 0.0001; KO vehicle vs. KO STZ: 0.1438 [0.1060, 0.18] (*p* < 0.0001); TA: 0.025 [0.013, 0.037], *p* < 0.0001; KO vehicle vs. KO STZ: 0.045 [0.033, 0.057] (*p* < 0.0001)) (Figure 2A,B). Thus, the decrease in GA and TA muscle weights caused by STZ was more pronounced in the KO group.

According to the results of the two-way ANOVA for EDL, the main effects of genotype (*p* = 0.012) and STZ (*p* < 0.0001) were significant, whereas the interaction effect between genotype and STZ was not significant (*p* = 0.15) (Figure 2C). For SOL, two-way ANOVA revealed that the main effect of STZ (*p* = 0.0008) was significant, but neither the main effect of genotype (WT vs. KO) (*p* = 0.44) nor the interaction between genotype and STZ was significant (*p* = 0.15) (Figure 2D). Consequently, among the groups, the weights of the GA and TA groups were the lowest among the KO groups. In both the GA and TA, the STZ-induced reduction in muscle weight was more pronounced in the KO mice than in the WT mice.

Two-way ANOVA revealed a significant interaction between time and group (WT vehicle, WT STZ, KO vehicle, and KO STZ) (*p* < 0.0001). Two weeks after STZ treatment, both WT and KO mice presented a reduction in limb grip strength (mean difference pre-post [95% CI]: WT vehicle, 3.0 [−12.3, 18.2]; WT STZ, 55.6 [21.2, 89.9] (*p* = 0.0046); KO vehicle, 4.6 [−15.2, 24.4]; and KO STZ, 85.6 [69.1, 102.1] (*p* < 0.0001)) (Figure 2E). Although no differences between groups were observed when the animals reached 18 weeks of age, two weeks post-STZ administration, the mean differences [95% CIs] between vehicle-treated WT-vehicle mice and WT-STZ mice, between WT-STZ mice and KO-STZ mice, and between KO-vehicle mice and KO-vehicle mice were 49.6 [0.9, 98.4] (*p* = 0.046), 71.40 [29.1, 113.7] (*p* = 0.0059), and 84.3 [51.9, 116.8] (*p* = 0.0003), respectively (Figure 2E). Consequently, STZ-treated KO mice presented the lowest grip strength.

Gene expression analysis via two-way ANOVA revealed a significant interaction effect (IGF1: *p* = 0.0015; Myostatin: *p* = 0.042; Atrogen: *p* < 0.0001). IGF-1 expression levels were greater in the KO mice than in the WT mice (*p* = 0.013), with a trend toward increased myostatin expression in the KO mice (*p* = 0.051) (Figure 2F–H). The administration of STZ resulted in decreased IGF-1 expression (*p* = 0.07, *p* < 0.0001) and increased atrogin expression (*p* = 0.0006, *p* < 0.0001) in both WT and KO mice (Figure 2F–H). No changes in myostatin mRNA expression were observed following STZ administration (Figure 2F–H).

In summary, when *Chrebp* deletion occurs in the context of insulin deficiency, decreases in muscle mass and grip strength are accelerated.

### 2.3. Insulin Deficiency Promotes Osteopenia in Chrebp Knockout Mice

#### 2.3.1. Bone Mineral Density and Stiffness

Next, we examined the effects of insulin deficiency and *Chrebp* deletion on bone density and bone strength.

**Figure 3:**
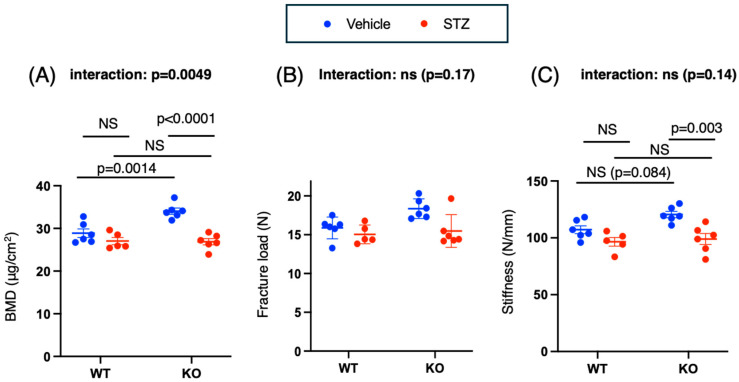
Effects of Chrebp deletion and insulin deficiency on BMD and stiffness. (**A**) Bone mineral density. Two-way ANOVA. Genotype (WT vs. KO): *p* < 0.0001; drug (STZ): *p* = 0.0075; genotype × drug interaction: *p* = 0.0049. STZ was administered intraperitoneally to 18-week-old WT and KO mice, and experiments were conducted two weeks later. During tissue removal, the bones were preserved and used in further experiments. The bone mineral density of the femur was measured via noninvasive dual-energy X-ray absorptiometry (DXA). The data are presented as the means ± standard errors of the means (SEMs) and were analyzed via two-way analysis of variance (ANOVA) with post hoc Tukey’s test for each group (vehicle: blue; STZ: red). *n* = 5–6, *p* < 0.05. (**B**) Stiffness (N/cm^2^). Two-way ANOVA. Genotype (WT vs. KO): *p* = 0.051; drug (STZ): *p* = 0.0004; genotype × drug interaction: *p* = 0.17. (**C**) Fracture load (N). Two-way ANOVA. Genotype (WT vs. KO): *p* = 0.037; drug (STZ): *p* = 0.010; genotype × drug interaction: *p* = 0.14. The stiffness and fracture load of the femur were measured via three-point bending tests. The data are presented as the means ± SEMs and were analyzed via two-way ANOVA with post hoc Tukey’s test for each group. Post hoc analyses were performed following two-way ANOVA exclusively for interactions that demonstrated statistical significance. *n* = 5–6, *p* < 0.05.

In terms of bone mineral density (BMD), the effect of the interaction between genotype and drug was significant (*p* = 0.004). The BMD of the animals in the *Chrebp*-deficient group was greater than that of the animals in the wild-type group (WT vehicle vs. KO vehicle: −5.2 [−8.4, −1.9], *p* = 0.0014) (Figure 3A). However, the administration of STZ significantly decreased the BMD only in the KO group (KO vehicle vs. KO STZ: 7.2 [3.9, 10.4], *p* < 0.0001). There was no significant difference in BMD between STZ-treated WT and KO mice (0.16 [−3.3, 3.6], *p* = 0.999) (Figure 3A). Therefore, the decrease in BMD due to STZ administration was greater in the KO mice than in the WT mice.

In the two-way ANOVA analysis of fracture load, both the main effect of genotype (*p* = 0.037) and the main effect of drug (*p* = 0.010) were statistically significant, whereas the interaction between genotype and drug was not significant (*p* = 0.17) (Figure 3B). In the two-way ANOVA evaluating stiffness, only the main effect of drug type (*p* = 0.004) reached statistical significance, whereas the main effects of genotype (*p* = 0.051) and the genotype × drug interaction (*p* = 0.17) did not achieve significance (Figure 3C). Consequently, the reduction in BMD induced by STZ was more pronounced in the KO group.

#### 2.3.2. Bone Structure Analysis by μCT

We investigated the effects of insulin deficiency and Chrebp deletion on bone structure via μCT.

**Figure 4:**
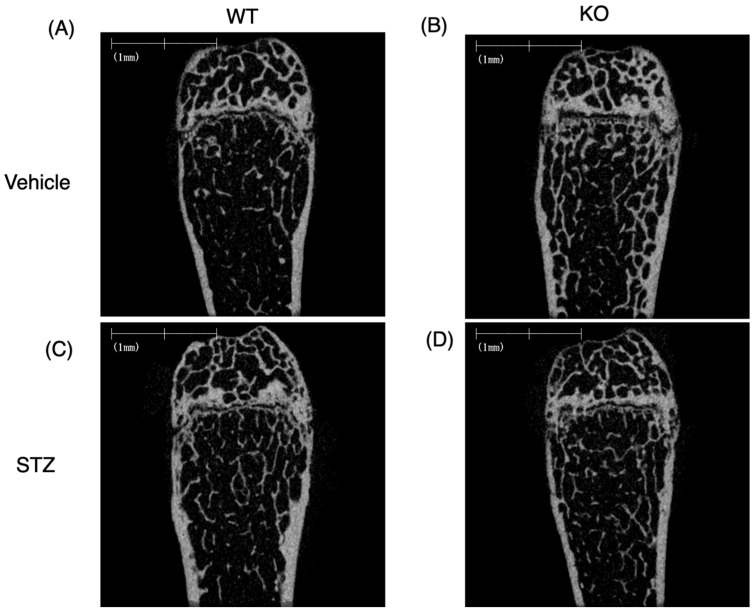

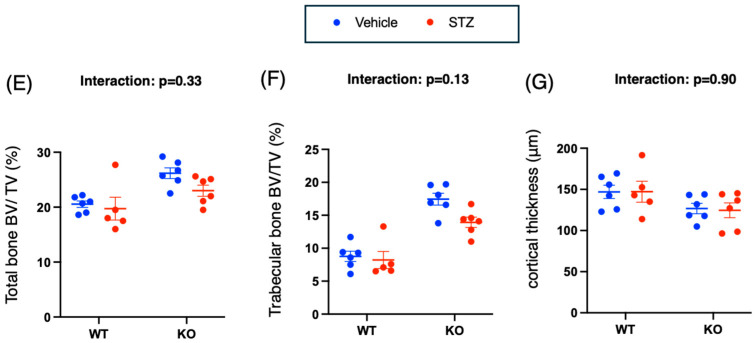
Effects of Chrebp deletion and insulin deficiency on the structural parameters of bone. Micro-CT images of (**A**) vehicle-treated wild-type (WT) mice, (**B**) vehicle-treated knockout (KO) mice, (**C**) STZ-treated WT mice, and (**D**) STZ-treated KO mice. (**E**) Total bone volume per total volume BV/TV (%). Two-way ANOVA. Genotype (WT vs. KO): *p* = 0.0015; drug (STZ): *p* = 0.11 genotype × drug interaction: *p* = 0.33. (**F**) Trabecular bone BV/TV (%). Two-way ANOVA. Genotype (WT vs. KO): *p* < 0.0001; drug (STZ): *p* = 0.042; genotype × drug interaction: *p* = 0.13. (**G**) Cortical thickness (μm). Two-way ANOVA. Genotype (WT vs. KO): *p* = 0.029; drug (STZ): *p* = 0.92; genotype × drug interaction: *p* = 0.90. BV/TV, cancellous bone BV/TV, and cortical thickness were measured via micro-CT. STZ was administered intraperitoneally to 18-week-old WT and KO mice, and experiments were conducted two weeks later. During tissue removal, the bones were preserved and used in further experiments. Micro-CT was performed according to the methods described in the methods section. The data are presented as the means ± standard errors (SEMs) and were analyzed via two-way analysis of variance (ANOVA) with post hoc Tukey’s test for each group (vehicle: blue; STZ: red). Post hoc analyses were performed following two-way ANOVA exclusively for interactions that demonstrated statistical significance. *n* = 5–6, *p* < 0.05. NS, not significant.

In the two-way ANOVA assessing total bone volume/tissue volume, the main effect of genotype was statistically significant (*p* = 0.0015), whereas the main effect (*p* = 0.11) and the genotype interaction (*p* = 0.33) did not reach statistical significance (Figure 4A–E). For BV/TV, the main effects of both genotype (*p* < 0.0001) and STZ (*p* = 0.042) were statistically significant, although the genotype interaction (*p* = 0.13) was not significant (Figure 4A–D,F). In the two-way ANOVA for cortical thickness, the main effect of genotype (WT vs. KO) was significant (*p* = 0.029), whereas the main effect of drug (*p* = 0.92) and the genotype interaction (*p* = 0.90) were not significant (Figure 4G).

In summary, although the BV/TV was greater in the KO vehicle group than in the WT vehicle group, the BV/TV of the STZ-KO group decreased to a level similar to that of the WT-KO group. Effects due to differences in genotype are observed for total and trabecular BV/TV, but the effect of STZ does not differ between WT and KO mice.

#### 2.3.3. Bone Structure Analysis of Microscopic Specimens

We also investigated the effects of insulin deficiency and *Chrebp* deletion on bone structure in histological sections.

First, bone volume and trabecular microarchitecture were examined via bone volume/tissue volume (BV/TV, the volume fraction of mineralized bone within the tissue volume, representing trabecular bone mass) and trabecular thickness (Tb.Th, the average thickness of individual trabeculae within the region of interest), trabecular number (Tb.N, the number of trabeculae per unit length, an index of trabecular connectivity), and trabecular separation (Tb.Sp, the mean distance between trabeculae, reflecting the degree of trabecular rarefaction).

**Figure 5:**
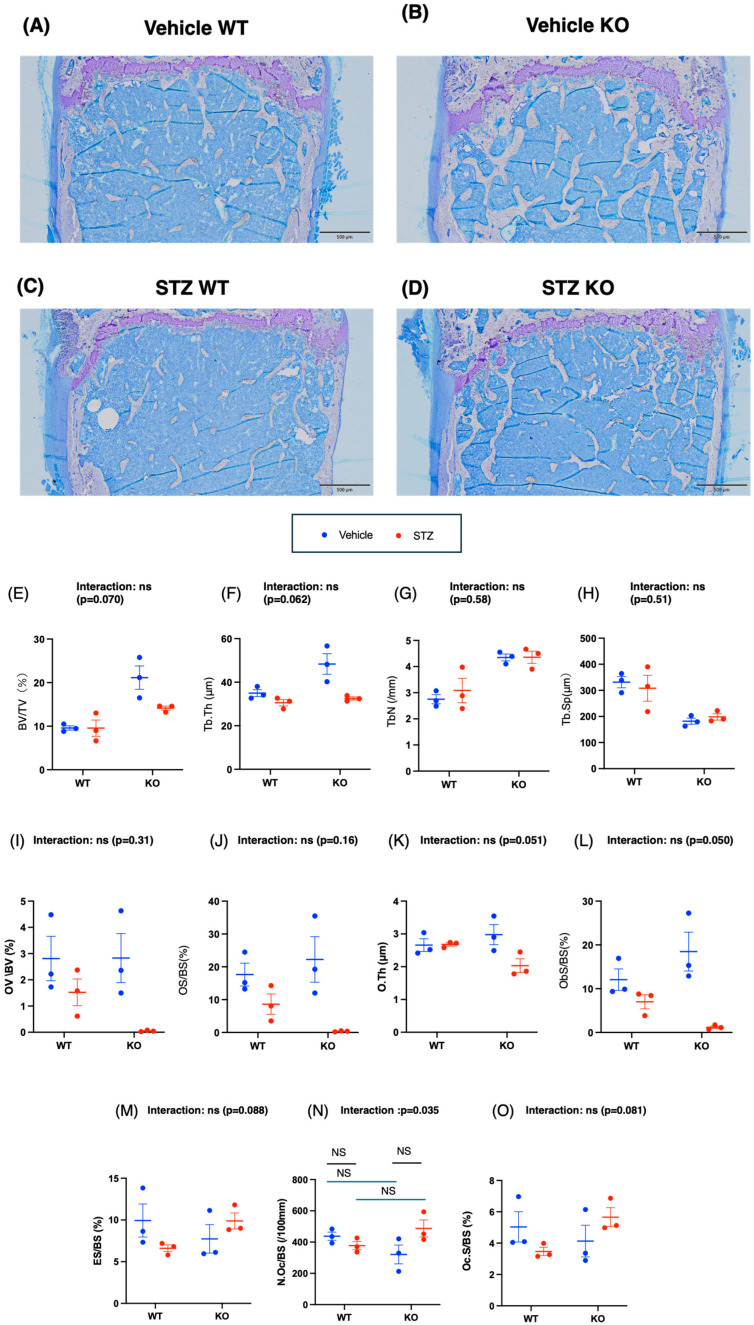
Effects of Chrebp deletion and insulin deficiency on bone structure, bone formation, and bone resorption. Representative images (magnification: 40×) of femur epiphyses from (**A**) the vehicle-treated WT group, (**B**) the vehicle-treated KO group, (**C**) the STZ-treated WT group, and (**D**) the STZ-treated KO group. Scale bar = 500 μm. (**E**) Bone volume (BV/TV) (%). Two-way ANOVA. Genotype (WT vs. KO): *p* = 0.019 drug (STZ): *p* = 0.067; genotype × drug interaction: *p* = 0.070. (**F**) Trabecular thickness (mm). Two-way ANOVA. Genotype (WT vs. KO): *p* = 0.019; drug (STZ): *p* = 0.0049; genotype × drug interaction: *p* = 0.062. (**G**) Number of trabeculae (mm). Two-way ANOVA. Genotype (WT vs. KO): *p* = 0.0010; drug (STZ): *p* = 0.56; genotype × drug interaction: *p* = 0.58. (**H**) Trabecular separation (µm). Two-way ANOVA. Genotype (WT vs. KO): *p* = 0.0018; drug (STZ): *p* = 0.91; genotype × drug interaction: *p* = 0.51. (**I**) Osteoid volume/bone volume (OV/BV) ratio; two-way ANOVA. Genotype (WT vs. KO): *p* = 0.0017; drug (STZ): *p* = 0.32; genotype × drug interaction: *p* = 0.31. (**J**) Osteoid surface/bone surface (OS/BS) ratio; two-way ANOVA. Genotype (WT vs. KO): *p* = 0.67; drug (STZ): *p* = 0.0061; genotype × drug interaction: *p* = 0.16. (**K**) Osteoid thickness (O.Th.), two-way ANOVA. Genotype (WT vs. KO): *p* = 0.46; drug (STZ): *p* = 0.059; genotype × drug interaction: *p* = 0.051. (**L**) Osteoblast surface/bone surface (Ob.S/BS), two-way ANOVA. Genotype (WT vs. KO): *p* = 0.91; drug (STZ): *p* = 0.0030; genotype (WT vs. KO) × drug interaction: *p* = 0.050. (**M**) Eroded surface/bone surface (ES/BS) two-way ANOVA. Genotype (WT vs. KO): *p* = 0.71; drug (STZ): *p* = 0.69; genotype (WT vs. KO) × drug (STZ) interaction: *p* = 0.088. (**N**) Number of osteoclasts/bone surface (N.Oc/BS). Two-way ANOVA. Genotype (WT vs. KO): *p* = 0.94; drug (STZ): *p* = 0.27; genotype (WT vs. KO) × drug (STZ) interaction: *p* = 0.035. (**O**) Osteoclast surface/bone surface (OC.S/BS) Two-way ANOVA. Genotype (WT vs. KO): *p* = 0.43; drug (STZ): *p* = 0.98; genotype × drug interaction: *p* = 0.081. The structural, dynamic, and cellular parameters were calculated and expressed according to the standard nomenclature. The data are presented as the means ± standard errors of the means and were analyzed via two-way analysis of variance (ANOVA) with post hoc Tukey’s test for each group (vehicle: blue; STZ: red). Post hoc analyses were performed following two-way ANOVA exclusively for interactions that demonstrated statistical significance. Post hoc analyses were performed following two-way ANOVA exclusively for interactions that demonstrated statistical significance. *n* = 3, *p* < 0.05. NS, not significant. The total tissue volume showed no biologically meaningful differences among the groups, indicating that the regions of interest were consistently defined. Blue and red indicate vehicle and STZ treatment, respectively.

Two-way ANOVA conducted on BV/TV indicated a significant main effect of genotype (WT vs. KO) (*p* = 0.0013). In contrast, the main effect of the drug (STZ) (*p* = 0.067) and the interaction effect between genotype and drug (*p* = 0.070) approached significance (Figure 5E). In a two-way ANOVA on Tb.Th, significant main effects were observed for both genotype (WT vs. KO) (*p* = 0.0019) and drug (STZ) (*p* = 0.0049). The interaction effect between genotype and drug was also marginally significant (*p* = 0.062) (Figure 5F). For Tb.N., two-way ANOVA revealed a significant main effect of genotype (WT vs. KO) (*p* = 0.0010), whereas the main effect of drug (STZ) (*p* = 0.56) and the interaction effect between genotype and drug (*p* = 0.58) were not significant (Figure 5G). Similarly, in a two-way ANOVA on Tb.Sp., the main effect of genotype (WT vs. KO) was significant (*p* = 0.0018), whereas the main effect of drug (STZ) (*p* = 0.91) and the interaction effect between genotype and drug (*p* = 0.50) were not significant (Figure 5H).

Thus, bone volume and trabecular microarchitecture are influenced by genotype (WT vs. KO). The main effect of the drug (STZ) was observed only in Tb.Th, while the BV/TV tended to decrease with STZ administration.

The bone formation and osteoid indices included the osteoid volume/bone volume (OV/BV, the proportion of unmineralized bone matrix (osteoid) relative to the total bone volume), and osteoid surface/bone surface (OS/BS, the percentage of the bone surface covered by the osteoid, indicating active bone formation prior to mineralization), and osteoid thickness (O.th., the average thickness of osteoid seams on the bone surface) were measured, and as indicators of osteoblast formation activity, the osteoblast surface/bone surface (Ob. S/BS, the percentage of the bone surface covered by osteoblasts, reflecting bone-forming activity) was measured.

Two-way ANOVA revealed that the main effect of STZ on OV/BV was significant (*p* = 0.017), whereas the main effects of genotype (WT vs. KO) (*p* = 0.31) and the interaction effect between genotype and drug (*p* = 0.30) were not significant (Figure 5I). Similarly, two-way ANOVA revealed that the main effect of STZ on OS/BS was significant (*p* = 0.0061), whereas the main effects of genotype (WT vs. KO) (*p* = 0.67) and the interaction effect between genotype and drug (*p* = 0.16) were not significant (Figure 5J). In the analysis of O.Th., the main effect of genotype (WT vs. KO) (*p* = 0.46) was not significant, but the main effect of drug (STZ) (*p* = 0.059) and the interaction effect between genotype and drug (*p* = 0.051) approached significance (Figure 5J). In the two-way ANOVA for ObS/BS, the main effect of STZ was significant (*p* = 0.0030), the main effect of genotype (WT vs. KO) (*p* = 0.91) was not significant, and the interaction effect between genotype (WT vs. KO) and drug (*p* = 0.050) approached significance (Figure 5J).

Therefore, parameters related to bone formation are affected by only the drug (STZ).

As a parameter of the bone resorption surface, the eroded surface/bone surface (ES/BS, the proportion of the bone surface showing evidence of resorption lacunae, representing bone-resorbing activity) was measured, and as an indicator of osteoclast activity, the number of osteoclasts/bone surface (N.OC/BS, the number of osteoclasts per unit of bone surface, serving as an index of osteoclast abundance) was measured. and osteoclast surface/bone surface (OC. S/BS, the percentage of the bone surface covered by osteoclasts, indicating the extent of active bone resorption, was measured.

In a two-way ANOVA on ES/BS, neither the main effect of genotype (WT vs. KO) (*p* = 0.71) nor the main effect of drug (STZ) (*p* = 0.69) was significant. However, the interaction effect between genotype and drug type was significant (*p* = 0.050, *p* = 0.088) (Figure 5J). A similar analysis of N.Oc/BS revealed a significant interaction effect between genotypes (*p* = 0.035) (Figure 5N). Nonetheless, there were no significant differences in the number of osteoclasts (N.Oc/BS/100 mm) among the groups (Figure 5N). For OC.S/BS, the main effects of genotype (*p* = 0.43) and drug (STZ) treatment (*p* = 0.98) were not significant, whereas the interaction effect between genotype and drug (STZ) treatment tended to be significant (*p* = 0.081, *p* = 0.088) (Figure 5J). Consequently, no significant differences were observed in ES/BS, the number of osteoclasts (N.Oc/BS/100 mm), or the osteoclast surface area (Oc.S/BS, %) among the groups (Figure 5M–O).

#### 2.3.4. mRNA Analysis of Bone

Finally, to examine the effects of insulin deficiency and Chrebp deletion on the expression of genes involved in bone formation (BMP2, Osx, and Opg) and bone resorption (Rankl and Sclerostin), we analyzed the mRNAs present in the bone samples.

**Figure 6:**
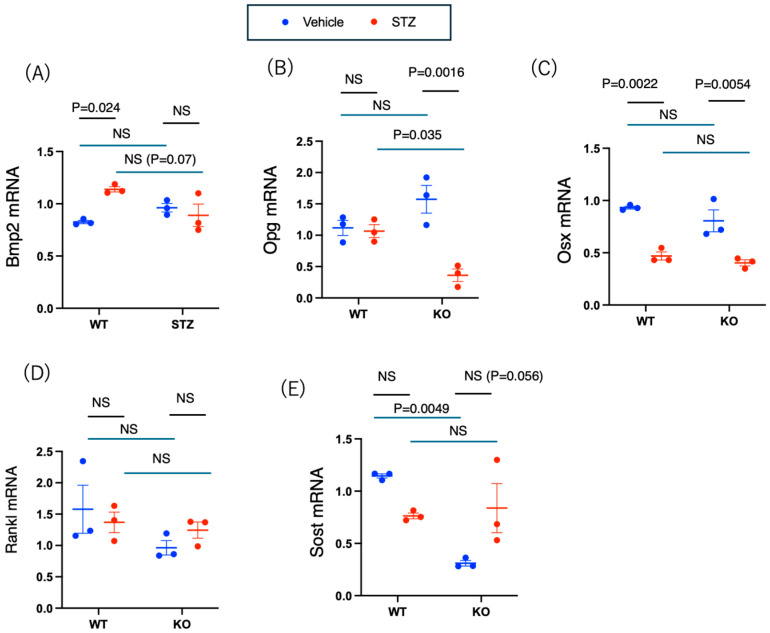
Effects of *Chrebp* deletion and a low-protein diet on bone formation and resorption-related mRNA expression. (**A**) Bmp2 mRNA levels. Two-way ANOVA. Genotype (WT vs. KO): *p* = 0.37; drug (STZ): *p* = 0.077; genotype × drug interaction: *p* = 0.012. (**B**) Opg mRNA levels. Two-way ANOVA. Genotype (WT vs. KO): *p* = 0.41; drug (STZ): *p* = 0.0024; genotype × drug interaction: *p* = 0.0038. (**C**) Osx mRNA levels. Two-way ANOVA. Genotype (WT vs. KO): *p* = 0.13; drug (STZ): *p* < 0.0001; genotype × drug interaction: *p* = 0.61. (**D**) Rankl mRNA levels. Two-way ANOVA. Genotype (WT vs. KO): *p* = 0.14; drug (STZ): *p* = 0.87; genotype × drug interaction: *p* = 0.31. (**E**) Sost mRNA levels. Two-way ANOVA. Genotype (WT vs. KO): *p* = 0.013; drug (STZ): *p* = 0.56; genotype (WT vs. KO): × drug (STZ) interaction: *p* = 0.0052. Femoral heads from WT and KO mice treated with vehicle or STZ for 12 weeks were isolated and ground in liquid nitrogen; RNA was subsequently isolated, and cDNA was synthesized. The mRNA levels were normalized to those of mouse Pol2 mRNA. The data are presented as the means ± standard errors of the means and were analyzed via two-way analysis of variance (ANOVA) with post hoc Tukey’s test for each group (vehicle: blue; STZ: red). Post hoc analyses were performed following two-way ANOVA exclusively for interactions that demonstrated statistical significance. Post hoc analyses were performed following two-way ANOVA exclusively for interactions that demonstrated statistical significance. *n* = 3, *p* < 0.05. NS, not significant.

A two-way ANOVA analysis of Bmp2 revealed a significant interaction effect between genotype and drug on BMP expression (*p* = 0.012). Bmp2 is crucial for the differentiation of stem cells into osteoblasts, which are essential for bone formation. The mRNA levels of bone morphogenetic protein 2 (Bmp2) in both the WT and KO groups did not differ following vehicle administration; however, a significant increase was observed exclusively in the WT group post-STZ administration (WT vehicle–WT STZ: −0.31 [−0.58, −0.045], *p* = 0.024; KO vehicle–KO STZ: 0.073 [−0.20, 0.34], *p* = 0.82) (Figure 6A). In a two-way ANOVA of Opg, a significant interaction effect between genotype and drug was identified (*p* = 0.0038). Osteoprotegerin (OPG) functions as an inhibitor, preventing RANKL from activating osteoclasts, thereby mitigating bone resorption and enhancing bone mass. Following STZ administration, a significant reduction in Opg mRNA was noted solely in the KO group (WT vehicle—WT STZ: 0.050 [−0.60, 0.70], *p* = 0.99; KO vehicle—KO STZ: 1.21 [0.56, 1.87], *p* = 0.016) (Figure 6B). The two-way ANOVA on Osterix (Osx) indicated that the main effect of genotype (WT vs. KO) (*p* = 0.13) was not significant, whereas the main effect of drug (STZ) (*p* < 0.0001) was significant, and the genotype × drug interaction (*p* = 0.61) was not significant (Figure 6C). Sclerostin, a protein associated with bone resorption and produced by osteocytes, inhibits the Wnt signaling pathway, which is crucial for osteoblast function. The receptor activator of nuclear factor-κB ligand (RANKL) on osteoblasts binds to its receptor (RANK) on pre-osteoclasts, facilitating bone resorption. A two-way ANOVA of Rankl mRNA demonstrated a significant interaction effect between genotype and drug on Sost (*p* = 0.013). However, no significant differences in Rankl mRNA expression were observed among the groups (Figure 6D). A two-way ANOVA of sclerostin (Sost) mRNA revealed a significant interaction effect between genotype and drug on sclerostin (Sost) (*p* = 0.0052). Sost mRNA expression levels were significantly reduced in the KO group compared to the WT group following vehicle treatment (*p* = 0.0049) (Figure 6E). These results suggest that insulin deficiency results in a reduction of Opg and Osx mRNAs, while Chrebp deletion leads to an increase in sclerostin mRNA. An increase in BMP mRNA due to STZ administration was observed only in the WT group, whereas the reduction in Opg induced by STZ was more pronounced in the KO group, and the changes in Sost induced by STZ also differed between the WT and KO groups.

## 3. Discussion

In this study, we investigated how a combined deficiency in insulin and glucose signaling affects muscles and bones. Deletion of Chrebp alone did not result in decreased muscle mass or strength in 20-week-old mice; however, in animals that had experienced insulin deficiency for only two weeks, it promoted decreases in both muscle mass and strength. Furthermore, although bone density and sclerostin levels increased after Chrebp deletion alone, bone density decreased to the level observed in wild-type mice when insulin was deficient. These findings suggest that both insulin and glucose play important roles in maintaining muscle and bone density.

In mice in which ChREBP was deleted, the weight loss caused by insulin deficiency was more pronounced than that in wild-type mice. The prevalence of sarcopenia is high in individuals with type 1 diabetes, a condition of insulin deficiency [37], and the finding that sarcopenia occurs when Chrebp deficiency occurs in an insulin-deficient state is new insight.

Body weight includes both fat mass and lean mass (muscle mass). Because the changes in epididymal fat weight in the KO and WT mice were similar, the decrease in muscle mass was particularly notable in the KO mice. In addition, muscle mass was reduced in the KO mice treated with STZ. In our previous study in which mice were fed a low-protein diet, we reported additive decreases in grip strength and muscle mass when a low-protein diet was combined with Chrebp deletion; we also reported an increase in myostatin levels and a decrease in IGF-1 levels in those animals, similar to the results observed in the Chrebp KO mice treated with STZ in the present study [23]. ChREBP has a paralog, MondoA, that also forms a complex with the Chrebp partner Mlx, and it indirectly suppresses fatty acid synthesis by inhibiting glucose uptake [38,39,40]. A point that was not investigated in the present study is the possibility that deletion of Chrebp enhances the effects of MondoA and thus increases the likelihood of a reduction in muscle mass and strength. A comparison of Mlx knockout mice (or MondoA knockout mice) with Chrebp knockout mice may be useful for investigating this hypothesis.

The present study demonstrated that bone mineral density (BMD) is influenced by genotype (WT vs. KO), with the KO group exhibiting greater BMD. Additionally, bone volume and trabecular microarchitecture are affected by genotype (WT vs. KO), which aligns with previous findings in *Chrebp* knockout mice [23]. Although the effects of genotype (WT vs. KO) on bone formation and bone resorption were not significant, only sclerostin levels were lower in KO vehicle-treated mice than in WT vehicle-treated mice. Sclerostin is a glycoprotein that is predominantly secreted by osteocytes and functions as a potent inhibitor of bone formation. Considering that sclerostin levels are lower in KO vehicle-treated mice than in WT vehicle-treated mice, the differentiation of osteoblasts and the formation of bone by these cells may be increased in KO vehicle-treated mice. Moreover, *Chrebp* overexpression in the MC3T3-E1 preosteoblast cell line has been reported to reduce the expression of the osteogenic genes Runx2 and ALP and ALP activity, whereas *Chrebp* knockdown has the opposite effects [35]. While there are reports that Chrebp is involved in bone formation, some studies have reported that ChREBP is present in and functions in macrophages. The loss of Chrebp may lead to the suppression of bone resorption. as it is in macrophages [41]. Finally, it is also possible that humoral factors from other organs (such as muscle and adipose tissue) may affect bone mineral density in bone tissue. Although it is not clear whether *Chrebp* is expressed in osteoblasts or osteoclasts, it has been found that systemic deletion of *Chrebp* leads to an increase in bone mass. We plan to analyze tissue (macrophage, muscle, and adipose tissue)-specific *Chrebp* knockout mice to further elucidate the role of Chrebp in bone formation and bone resorption.

Under insulin-deficient conditions, a reduction in the expression of bone formation markers and Osx mRNA was observed. As reported by Yang J et al., insulin promotes the differentiation of the osteoblastic cell line MG-63, but the induction of Osx by insulin is suppressed when ERK, which acts downstream of the insulin signal, is inhibited [42]. Thus, insulin defects affect BMD levels. Since changes in gene expression differ under conditions of insulin blockade and glucose signaling blockade, it is possible that insulin and glucose regulate bone formation and resorption through different mechanisms.

A decline in musculoskeletal function has been reported in type 1 diabetes patients [39,40,41,42,43,44,45]. Older age and lower BMI are the major determinants of sarcopenia in T1D patients, but conflicting data concerning diabetes duration, glucose control, and dietary and physical exercise behaviors are available. Additionally, another study revealed that among 62 patients with type 1 diabetes (66% females, mean age 38 ± 14 years, body mass index (BMI) 24.9 ± 4.7 kg/m^2^), the frequency of sarcopenia was 8% and that of dynapenia was 23%, with higher HbA1c levels observed in the sarcopenia group. Bone fragility is also known to occur in type 1 diabetes. People with type 1 diabetes have lower bone mineral density (BMD) and greater fracture risk than individuals without diabetes do (more than five times for hip fractures and two times for nonvertebral fractures) [43]. Poorer glycemic control, AGE accumulation, and kidney disease are independent risk factors for lower hip BMD in older adults with type 1 diabetes. A state in which both insulin signaling and glucose signaling are reduced, as observed in the STZ-administered *Chrebp* knockout mice in this study, might be similar to a clinical situation in which insufficient insulin is administered and only blood glucose is reduced, for example, with the use of SGLT2 inhibitors. Previously, we encountered a patient (in this case, with type 2 diabetes) who, despite decreased insulin secretion necessitating insulin therapy, was prescribed an SGLT2 inhibitor [44]. In this patient, there was a marked decrease in subcutaneous fat, visceral fat, and muscle mass, along with weight loss and reduced muscle strength. Although bone mineral density was not measured, given the low body weight, a decrease in bone mineral density was also expected. Another study reported that 36 patients with type 1 diabetes were given dapagliflozin 5 mg/day, and the skeletal muscle mass index was evaluated before and after treatment. In nonobese patients, a decrease in the skeletal muscle index was observed [45]. Therefore, in cases where SGLT2 inhibitors are used in conditions with decreased insulin secretion, including type 1 diabetes, it is necessary to pay attention not only to blood sugar but also to changes in body composition, including muscle mass and bone mineral density [46].

The present study demonstrated that a lack of insulin and glucose led to decreased muscle strength, reduced muscle mass, and decreased bone density only two weeks after STZ treatment. Compared with humans, mice have faster bone metabolic turnover and a shorter bone remodeling cycle; as a result, mice exhibit more rapid changes in bone density and bone strength than humans do [47]. For example, mouse cancellous bone turnover is approximately 0.7% per day, with remodeling completing in approximately two weeks, whereas human cancellous bone turnover is approximately 0.1% per day, and 6–9 months are required to complete a single remodeling cycle [47]. Considering the relative turnover in humans and mice, it should be assumed that bone density begins to decrease within a relatively few years in patients with type 1 diabetes. Therefore, follow-up data on body composition, grip strength, muscle mass, and bone mineral density may be necessary even for young individuals with type 1 diabetes mellitus.

The present study has several limitations. Along with elevated blood glucose levels, a tendency toward dehydration was observed. In fact, when blood samples were taken, the blood of the STZ-treated mice appeared to be more viscous. Although we were unable to measure electrolytes or body water content, in humans, sustained blood glucose levels exceeding 600 mg/dL can lead to a condition known as hyperglycemic hyperosmolar syndrome [48], which is accompanied by dehydration. Although we did not assess electrolyte or body water levels, the STZ-treated mice were considered to have a tendency toward dehydration. Because significant hyperglycemia was observed in the STZ-treated mice, dehydration may explain the observed reductions in muscle strength, muscle mass, and bone density. Dehydration itself can also be involved in decreasing muscle strength and bone density [49,50,51,52]. The blood glucose levels of the animals in the STZ-treated WT and KO groups were similar. Muscle weakness and decreased bone density due to STZ itself may be influenced to some extent by dehydration in both STZ-treated WT and STZ-treated KO mice. Moreover, other potential effects of STZ itself, in addition to its ability to induce hyperglycemia, cannot be entirely ruled out. However, any residual effects of STZ were considered minimal because two weeks elapsed after administration. Additionally, in this study, it was not possible to compare glucose tolerance between the KO and STZ-treated KO mice. The reason is that tests such as the glucose tolerance test cannot be conducted due to hyperglycemia. Since *Chrebp* deficiency causes impaired glucose tolerance, it is possible that STZ-treated KO mice have higher blood glucose levels than KO mice do. Glucose tolerance cannot be evaluated via random blood glucose measurements. While not common, tests such as HbA1c—if they could be performed on mice—might provide useful reference information. However, according to the results of the two-way ANOVA, the genotype × drug (STZ) interaction had a p value of 0.054 and was not significant; thus, there was no substantial difference between the WT and KO groups.

Moreover, in the present study, the expression of *Chrebp* in bone tissue was not detected. Although we attempted area staining, it could not be detected due to sensitivity issues. Similarly, as previously reported, *Chrebp* mRNA was also not detected in bone tissue. However, according to the Human Atlas, the expression of *Chrebp* has been confirmed in bone macrophages (corresponding to osteoclasts) [53]. Furthermore, the impact of Chrebp on glucose and lipid metabolism in osteoblasts and osteoclasts remains unexplored. In osteoblasts, insulin facilitates glucose uptake and glycolysis; in osteoblast-specific insulin receptor knockout mice, glycolysis in osteoblasts is inhibited, leading to diminished bone formation and reduced bone mass [54]. There are few studies concerning Chrebp and the glycolytic or fatty acid synthesis pathways in osteoblasts. During osteoblast differentiation, the expression of GLUT1, HK2, PFKFB3, and LDHA is upregulated, enhancing glycolytic activity and increasing lactate production, akin to the Warburg effect [55]. As cells mature into osteoblasts or osteocytes, glycolysis decreases, whereas the role of mitochondrial OXPHOS becomes more prominent [55]. Fatty acids are sourced from the surrounding bone marrow adipose tissue, with some derived through de novo lipogenesis; however, the contribution of de novo lipogenesis to newly synthesized lipids in vivo is reportedly lower than that in adipose tissue [56]. Conversely, reports indicate that *Chrebp* is expressed in human osteoclasts. Additionally, in osteoclasts, fatty acid synthase is induced under RANKL stimulation [57], and the inhibition of fatty acid synthesis enzymes suppresses osteoclast differentiation and reduces bone resorption [58]. For the above reasons, we plan to investigate this further using macrophage-specific knockout mice in the future. Finally, we used STZ as a model of insulin-deficient diabetes. STZ can cause acute kidney and liver damage, but theoretically, such damage can occur within a few days after administration [59,60]. Therefore, by observing two weeks after the peak, we avoided the effects of kidney and liver injury [52]. No atrophy of the liver or kidneys was found at dissection, and we did not use doses exceeding the standard; thus, the observed effects are considered to be due to diabetes. Although crossing with Akita mice or NOD mice is also a possible method, since diabetes develops in younger generations in those models, they do not match the purpose of this experiment; thus, we used the STZ method.

In this study, blockage of both insulin signaling and glucose signaling led to muscle atrophy and decreased bone density. Since it is difficult to detect the expression of *Chrebp* in bone, it is necessary to clarify the role of Chrebp in bone tissue by using cell type-specific mice, such as macrophage-specific *Chrebp* knockout mice. In addition, to elucidate the involvement of the bone–muscle–adipose tissue axis, adipose tissue-specific *Chrebp* knockout mice and skeletal muscle-specific *Chrebp* knockout mice need to be analyzed. In summary, in the context of insulin deficiency, the inhibition of Chrebp may contribute to the development of sarcopenia and bone loss.

## 4. Materials and Methods

### 4.1. Materials

Streptozocin was purchased from Merck/Millipore Sigma (Burlington, MA, USA). A mouse/rat insulin ELISA kit (M1108) was purchased from the Morinaga Institute of Biological Science (Yokohama, Japan).

### 4.2. Animals

The animal experiments were performed in accordance with the guidelines set forth in the National Institutes of Health Guide for the Care and Use of Laboratory Animals (NIH publications No. 8023, revised 1978). All animal care was approved by the Fujita Health University Animal Care and Use Committee (Approval Nos. APU22029 on 4 January 2022; APU22029-MD1 on 19 May 2022; APU22029-MD2 on 4 January 2024; APU22029-MD3 on 11 June 2024), Toyoake, Japan. The mice were housed at 23 °C with a 12-h light/dark cycle. Wild-type mice were obtained from SLC (Shizuoka, Japan). KO mice were backcrossed for 10 generations on a C57BL/6J background (14–16). WT and KO mice were housed separately, with three mice per cage. The mice had free access to water and were fed an autoclaved CE-2 diet (CLEA Japan, Inc., Tokyo, Japan).

The animal groups and experimental design were as follows.

A total of 48 mice were used in this study. The animals were 8 weeks old, male and randomly assigned to each experimental group (n = 12 per group). Randomization was performed via a simple random allocation method. Investigators responsible for [outcome measurements (bone measurement)/histological evaluation (bone)] were blinded to the group assignments throughout the study. The mice were monitored at least once daily throughout the study. Humane endpoints were predefined to minimize animal suffering.

The animals were humanely euthanized if they met any of the following conditions:

(1) >20% loss of body weight compared with baseline;
(2) A marked reduction in food or water intake for more than 24–48 h;
(3) Severe lethargy or inability to ambulate;
(4) persistent signs of distress, such as piloerection, dehydration, or respiratory difficulty;
(5) Experimental-specific complications that could not be alleviated.

Euthanasia was performed via isoflurane anesthesia followed by cervical dislocation.

### 4.3. Streptozocin Administration

STZ dissolved in citrate buffer (pH 4.5) was administered intraperitoneally to 18-week-old male WT and KO mice as a single dose of 200 mg per kg body weight [28]. As a control (vehicle), only citrate buffer was administered. Blood glucose levels were measured at two days and at two weeks after STZ administration; blood glucose levels greater than 300 mg/dL were considered indicative of a diabetic state. At week 18, vehicle and STZ treatments were administered, and the number of mice in the experiments at week 20 was as follows: *n* = 11 (WT vehicle), *n* = 9 (WT STZ), *n* = 10 (KO vehicle), and *n* = 9 (KO STZ).

### 4.4. Body and Tissue Weight Measurements

At 20 weeks of age (two weeks after STZ administration), the mice (*n* = 9–11) were weighed, and blood was collected. After euthanasia by cervical dislocation, the tissues were removed, and the organ weights were measured. Bones (femoral heads) and muscles for mRNA analysis were preserved by freezing in liquid nitrogen.

### 4.5. Measurement of Blood Glucose, Insulin, and 3-Hydroxybutyrate Concentrations

Blood samples (*n* = 9–11 per group) were obtained from the tail veins of the mice. Blood samples were collected between 9:00 and 11:00 a.m. under ad. lib. fed conditions. Plasma glucose levels (mg/dL) and 3-hydroxybutyrate (3-OHBA) levels (mM) were measured via a FreeStyle Precision Neo Meter (Abbott, Chicago, IL, USA). Plasma insulin levels (ng/dL) were measured via a mouse/rat insulin kit (Morinaga Institute of Biological Science, Yokohama, Japan).

### 4.6. Muscle Weight and Limb Grip Strength Measurement

Muscle weight (g) and limb grip strength (*n* = 9–11, each) were measured according to previously described methods [27]. The weights of the tibialis anterior (TA), gastrocnemius, soleus, and extensor digitorum longus muscles were measured [27]. Limb grip strength tests were performed via a digital force meter (GPM-100B, MELQUEST, Toyama, Japan) [27]. The mouse was held in the grip, its tail was manually pulled backward, and the maximum grip force applied before the grip was released was measured [27]. The mouse gripped the wire mesh with all four limbs. Grip strength (g) is presented as the average of five measurements per mouse at 18 weeks (Pre) and 20 weeks (Post).

### 4.7. Bone Mineral Density Measurement

Bone mineral density measurements (*n* = 6 each) were performed at the Kureha Special Laboratory (Tokyo, Japan) as described in our previous study [27]. Bone mineral density (μg/cm^2^) was measured via an iNSiGHT noninvasive dual-energy X-ray absorptiometry (DXA) system (OsteoSys Inc., Seoul, Republic of Korea) (Figure 1).

### 4.8. Three-Point Bending Tests

Three-point bending tests (*n* = 6 each) were performed on mouse femurs to assess bone strength (the maximum load at which the bone breaks (fracture load (N)) and flexibility (stiffness (N/cm^2^)) [27]. The use of this test on long bones ensures that the response is predominantly flexural. Three-point bending tests were performed as described previously at the Kureha Special Laboratory [27]. Femurs were isolated from 30-week-old male mice, wrapped with Kimwipes dipped in saline, and stored in a −60 °C freezer. They were then thawed, and an MZ-500D electromechanical testing machine (Maruto Testing Machine Co., Tokyo, Japan) was used to apply a load vertically to the midshaft with a constant displacement rate of 2 mm/min until fracture occurred. A support span of 6 mm was used.

### 4.9. Microcomputed Tomography Analysis

Quantitative microcomputed tomography (µCT) analysis (*n* = 6, each) was performed via a µCT system (Scan Xmate-L090H; Comscantechno Co., Ltd., Yokohama, Japan) at the Kureha Special Laboratory (Figure 1) [27]. The morphometric parameters of the femurs were calculated through three-dimensional analysis of the data obtained from the scanned slices. Femoral trabecular bone parameters (total bone volume/tissue volume (BV/TV) (%), cancellous bone BV/TV (%), and cortical thickness (μm)) were calculated for the distal femoral metaphysis of the animals at 30 weeks of age.

### 4.10. Bone Histomorphometric Analysis

Bone histomorphometric analysis (*n* = 3 each) was performed at the Kureha Special Laboratory as described in our previous studies. The femurs of WT and KO mice were harvested and fixed in 10% formalin. The fixed bones were dehydrated by passage through a graded ethanol series, infiltrated and embedded in a mixture of methyl methacrylate and 2-hydroxyethyl methacrylate (both from Fujifilm Wako Pure Chemical, Osaka, Japan). Undecalcified 3-μm-thick sections were prepared via a microtome (Leica RM 2255; Leica Biosystems, Nussloch, Germany) [27]. Consecutive sections were stained with 0.05% toluidine blue (pH 7.0) for visualization of osteoids, osteoblasts, and osteoclasts. Bone histomorphometric analysis of the femurs was performed under 200× magnification within a 0.75 mm high × 0.7 mm wide region located 300 μm from the growth plate via Bone Histometry RT camera analysis software (version: Bone60 v1.26b; System-Supply, Nagano, Japan). Twenty-micron cross-sections from the mid-diaphyses of the femurs were used for bone histomorphometric analysis of cortical bone. The structural, dynamic, and cellular parameters were calculated and expressed according to the standard nomenclature [27]. The following parameters were measured: structural parameters (bone volume per total volume (BV/TV), trabecular number (Tb.N), and trabecular separation), bone formation parameters (osteoid volume/bone volume (OV/BV), osteoid surface/bone surface (OS/BS), osteoblast surface/bone surface (Ob.S/BS), and osteoid thickness (O.Th)), and bone resorption markers (osteoclast surface/bone surface (Oc.S/BS), osteoclast number/bone surface (N.Oc/BS), and eroded surface/bone surface (ES/BS)).

### 4.11. RNA Isolation and Quantitative Reverse Transcription–PCR

mRNA isolation, cDNA synthesis, and quantitative reverse-transcription PCR (qRT–PCR) analysis were performed as previously described [27]. Briefly, RNA was extracted from 100 mg of frozen muscle or proximal femur pooled from three mice per sample via the RNeasy Plus Universal Kit^TM^ (Qiagen, Hilden, Germany). Femur and muscle samples were ground in a mortar under liquid nitrogen before RNA extraction. cDNA was synthesized via a high-capacity cDNA Reverse Transcription Kit (Thermo Fisher Scientific, Waltham, MA, USA). Gene expression levels were evaluated in three muscle and bone tissue samples. Relative quantification of the mRNA levels was performed with reference to a standard curve. RNA polymerase 2 (Pol2) was used as the reference gene. Real-time PCR (RT–PCR) was performed with an ABI PRISM9700 instrument (Thermo Fisher Scientific, Waltham, MA, USA). The primers used for RT–PCR were the same as those used in our previous study [14,27].

### 4.12. Statistical Analysis

The data are presented as the means ± SEs. Statistical analysis was performed via GraphPad Prism 10 (GraphPad Software, Boston, MA, USA). The data were analyzed via two-way ANOVA with post hoc Tukey’s test for each group. *p* < 0.05 was considered to indicate statistical significance. For glucose, insulin, 3-hydroxybutyric acid (3OHBA), body weight, epididymal fat weight, muscle weights (GA, TA, EDL, and SOL), muscle IGF-1, myostatin, and atrogin-1 mRNA levels, as well as bone parameters (BMD, fracture load, stiffness, total BV/TV, trabecular BV/TV, cortical thickness, Tb.Th, Tb.N, Tb.Sp, OV/BV, OS/BS, O.Th, Ob.S/BS, ES/BS, N.Oc/BS, and Oc.S/BS), two-way ANOVA was performed with Genotype (WT vs. KO) and Drug (STZ treatment) as the two factors to evaluate the main effects and their interaction. For body weight and grip strength, two-way ANOVA was performed with Time (Pre (18 weeks) vs. Post (20 weeks)) and groups (WT vehicle, WT STZ, KO vehicle, and KO STZ) as the two factors to evaluate the main effects and their interaction. When a significant interaction was detected, post hoc comparisons were conducted using Tukey’s multiple comparisons test.

## Data Availability

All of the datasets generated during and/or analyzed during the present study are not publicly available; however, they are available from the corresponding author upon reasonable request.
